# Admission high‐sensitivity C‐reactive protein levels improve the Grace risk score prediction on in‐hospital outcomes in acute myocardial infarction patients

**DOI:** 10.1002/clc.23749

**Published:** 2022-01-23

**Authors:** Xiao Long Lin, Hao Xuan Sun, Fan Qi Li, Jin Yang Zhao, Dong Hui Zhao, Jing Hua Liu, Qian Fan

**Affiliations:** ^1^ Department of Cardiology, Beijing An Zhen Hospital Capital Medical University, Beijing Institute of Heart, Lung, and Blood Vessel Diseases Beijing Chaoyang China

**Keywords:** acute myocardial infarction, Grace risk score, high‐sensitivity C‐reactive protein, in‐hospital outcomes

## Abstract

**Background:**

Acute myocardial infarction (AMI) is the main cause of death and disability in cardiovascular and cerebrovascular diseases. Both the Global Registry of Acute Coronary Events (Grace) score and high‐sensitivity C‐reactive protein (hs‐CRP) were associated with prognosis in patients with AMI. However, whether the addition of the hs‐CRP to Grace risk score could improve the predictive power of Grace risk score on the prognosis of patients with AMI is unclear.

**Hypothesis**: We hypothesized that the inclusion of hs‐CRP in the Grace risk score could improve the ability to correctly distinguish the occurrence of in‐hospital outcomes.

**Methods:**

We retrospectively enrolled 1804 patients with AMI in the final analysis. Patients were divided into four groups by hs‐CRP quartiles. The relation between hs‐CRP and Grace risk score was analyzed by Spearman rank correlation. Logistic regression was used to identify independent risk factors. The predictive value of hs‐CRP add to Grace risk score was evaluated by *C*‐statistic, net reclassification improvement (NRI), integrated differentiation improvement (IDI), calibration plot, and decision curve analysis.

**Results:**

The hs‐CRP and Grace risk score had a significantly positive correlation (*r* = .191, *p* < .001). hs‐CRP combined with Grace risk score could improve the ability of Grace risk score alone to correctly redistinguish the occurrence of in‐hospital outcome (*C*‐statistic = 0.819, *p* < .001; NRI = 0.05956, *p* = .007; IDI = 0.0757, *p* < .001).

**Conclusion:**

Admission hs‐CRP level was a significant independent risk factor for in‐hospital outcomes in patients with AMI. The inclusion of hs‐CRP in the Grace risk score could improve the ability to correctly distinguish the occurrence of in‐hospital outcomes.

## 1 INTRODUCTION

Acute myocardial infarction (AMI) is that the most serious form of coronary heart disease. About 550 000 individuals in the United States experience an AMI for the primary time every year.^1^ By 2030, the number of AMI patients in China will reach 23 million.^2^ In addition to the high incidence of AMI, it is the main cause of death and disability in cardiovascular and cerebrovascular diseases. Therefore, for patients with AMI, risk stratification is very important, especially identifying early adverse outcome risk.

To identify high‐risk patients, Current guidelines recommend the Global Registry of Acute Coronary Events (Grace) score, which has been proved and widely used as a tool for risk stratification of AMI.^3^ The Grace risk score system combines some clinical and biological variables and scores these variables to obtain an ultimate score, Patients were divided into low‐risk, medium‐risk, and high‐risk groups based on the ultimate score to predict their risk of adverse outcomes. This Grace risk score model only included two biological indicators: serum creatinine (SCr) and troponin, but some biological indicators closely related to myocardial infarction were not included. Therefore, whether the effect of Grace risk score on the prognosis of patients with AMI can be further improved by combining with these important biomarkers is unclear.

Inflammation is one of the mechanisms leading to AMI,^4^ high‐sensitivity C‐reactive protein (hs‐CRP) as a biomarker of inflammation has been proven to be an important risk factor for cardiovascular disease,^5^ It has also been shown to be associated with prognosis in patients with myocardial infarction.^6^ The relationship between hs‐CRP and the Grace risk score is not clear. The purpose of this study was to investigate the relationship between admission hs‐CRP level and in‐hospital outcome and whether the addition of the hs‐CRP to Grace risk score could improve the predictive power of Grace risk score on the prognosis of patients with AMI.

## 2 METHODS

### 2.1 Study population

The present study is a single‐center, retrospective observational cohort study. From January 2019 to December 2019, 1804 consecutive patients who were diagnosed with AMI performed percutaneous coronary intervention at Beijing Anzhen hospital were enrolled. The diagnosis of AMI must be based on at least two of the following three criteria: (1) a clinical history of ischemic chest pain, (2) dynamic evolution of electrocardiography, (3) dynamic changes of serum myocardial marker concentration in myocardial necrosis. The only exclusion criteria were the lack of hs‐CRP laboratory value at admission. The present study was performed by the Helsinki Declaration of Human Rights (2000) and approved by the Clinical Research Ethics Committee of Beijing An Zhen Hospital, Capital Medical University. Written informed consent was obtained from all patients.

### 2.2 Demographic and clinical data

Patients' data of demographic, clinical, and angiographic characteristics were collected from Beijing An Zhen Hospital's medical information recording system.

The serum hs‐CRP and the routine hematology, biochemical parameters were collected on admission and were measured by standard laboratory methods in the central lab of Beijing Anzhen Hospital.

Admission assessment indicators included in the Grace risk scoring model were obtained at hospital admission (age, heart rate, systolic blood pressure (BP), SCr level, Killip class, ST‐segment deviation, elevated cardiac enzymes, and cardiac arrest). The Grace risk score was calculated according to the Grace risk calculator (https://www.outcomes-umassmed.org/grace).

### 2.3 Definitions

Malignant arrhythmia was defined as the arrhythmia that causes hemodynamic disturbance in a short time, leading to syncope or even sudden death, including ventricular fibrillation, ventricular tachycardia, third‐degree atrioventricular block, and so forth. The mechanical complication was defined as a complication of anatomical changes in the heart after myocardial infarction, including septal perforation, rupture of the papillary muscle of the mitral valve, and rupture of the heart. Cardiogenic shock was defined as a severe acute peripheral circulatory failure resulting from a significant decrease in cardiac output due to extreme cardiac dysfunction. Bleedings events were defined using the Bleeding Academic Research Consortium (BARC) classifications.^7^

### 2.4 In‐hospital outcomes

The in‐hospital outcomes were the composite of death, malignant arrhythmia, mechanical complication, congestive heart failure (HF), cardiogenic shock, thrombosis, bleeding, stroke.

### 2.5 Statistical analysis

Continuous variables were presented as the mean ± standard deviation is consistent with a normal distribution, otherwise as to the median and interquartile range (IQR). Categorical variables were expressed as numbers and percentages. One‐way analysis of variance or Mann–Whitney *U* test was used to analyze differences in continuous variables. The Pearson *χ* ^2^ test, Fisher's exact test, or the Cochran–Armitage Trend test was used to analyze categorical variables. The correlation between the Grace risk score and traditional hs‐CRP was evaluated by Spearman correlation analysis. The admission values of hs‐CRP were divided into four quartiles to stratify the incidence rates of in‐hospital adverse events. Univariate and multivariate logistic regression was used to estimate the in‐hospital adverse events. The analysis results were presented by odds ratios and 95% confidence intervals (CIs).

Two logistic regression models were established: one is Grace risk score alone, the other is to add hs‐CRP as a continuous variable to Grace risk score to form a new risk prediction model. *C*‐statistic was calculated by receiver operating characteristic analysis, which reflects the discrimination of the model. Compare to the area under the curve (AUC) from the two models was tested by Delong's test.^8^ The Calibration plot was used to evaluate the calibration degree of the models. The decision curve analysis assesses the clinical benefit of the model. The net reclassification improvement (NRI) and integrated differentiation improvement (IDI) risk models were used to compare with the traditional Grace risk score model to evaluate whether the new risk model can correctly reclassify the in‐hospital outcome, as described by Pencina et al.^9^ Data were analyzed by IBM SPSS statistics 24 and R software. For all comparisons, two‐sided probability values less than .05 indicated statistical significance.

## 3 RESULTS

### 3.1 Baseline characteristics of patients

A total of 2092 consecutive patients confirmed AMI included in this study, 1804 had information about admission serum hs‐CRP and were enrolled in the final analysis. Among the 1804 patients, 393 patients occurred in‐hospital outcomes. All patients were divided into four teams by quartiles of serum hs‐CRP levels. The baseline characteristics of patients were presented in Table 1. The mean age was 59 years (IQR: 51–67), 80.3% were men. The proportion of patients with ST‐segment elevation myocardial infarction (STEMI) and non‐ST‐segment elevation myocardial infarction were 52.3% and 47.7%, respectively. Patients in higher hs‐CRP levels had higher heart rate, Grace risk score, white blood cell (WBC) count, SCr, fasting blood glucose (FBG), total cholesterol, low‐density lipoprotein cholesterol, CK‐MB, hs‐TNI, IIb/IIIa receptor antagonists, furthermore as lower left ventricular ejection fraction (LVEF), hemoglobin, and high‐density lipoprotein cholesterol.

**Table 1:** Baseline clinical characteristics of patients

	Serum hs‐CRP, mg/L				
Characteristics	Q1 (*n* = 453)	Q2 (*n* = 449)	Q3 (*n* = 451)	Q4 (*n* = 451)	*p*‐Value
Age (years)	59 (51–66)	58 (50–66)	59 (50–67)	60 (50–67)	.352
Male sex, *n* (%)	368 (81.2)	358 (79.7)	355 (78.7)	367 (81.4)	.709
BMI (kg/m^2^)	25.5 (23.6–27.7)	26.1 (24.0–28.4)	26.0 (24.1–28.4)	25.9 (23.7–30.5)	.018
Systolic BP (mmHg)	126 (117–135)	125 (117–135)	125 (114–136)	122 (110–142)	.023
Diastolic BP (mmHg)	75 (70–81)	75 (70–80)	76 (69–84)	72 (68–80)	.043
Heart rate (bpm)	70 (65–77)	70 (65–77)	71 (66–80)	72 (66–84)	<.001
Hypertension, *n* (%)	260 (57.4)	282 (62.8)	281 (62.3)	263 (58.3)	.234
Diabetes, *n* (%)	136 (30.0)	148 (33.0)	144 (31.9)	135 (29.9)	.708
Dyslipidemia, *n* (%)	208 (45.9)	207 (46.1)	199 (44.1)	198 (43.9)	.155
Previous or current smoking, *n* (%)	278 (61.4)	264 (58.8)	281 (62.3)	287 (63.6)	.497
Previous MI, *n* (%)	78 (17.2)	62 (13.8)	69 (15.3)	69 (15.3)	.566
Previous PCI, *n* (%)	40 (8.8)	31 (6.9)	31 (6.9)	38 (8.4)	.581
Previous CABG, *n* (%)	4 (0.9)	4 (0.9)	6 (1.3)	8 (1.8)	.567
Previous stroke, *n* (%)	48 (10.6)	54 (12.0)	36 (8.0)	57 (12.6)	.114
LVEF (%)	60 (54–65)	59 (53–63)	58 (52–63)	55 (49–66)	<.001
Grace score	108 (92–125)	109 (91–128)	114 (98–130)	120 (102–141)	<.001
Laboratory values at hospital admission					
WBC count (×10^9^/L)	7.20 (6.07–8.78)	7.75 (6.53–9.54)	8.06 (6.64–10.02)	8.85 (7.18–10.59)	<.001
Hemoglobin (g/L)	146 (136–156)	147 (135–156)	143 (132–152)	142 (129–152)	<.001
Platelet count (×10^9^/L)	222 (186–260)	228 (189–270)	236 (190–279)	224 (185–268)	.014
SCr (mmol/L)	72.90 (63.90–83.85)	74.30 (65.00–85.45)	74.50 (64.20–85.70)	76.40 (65.30–89.70)	.014
eGFR (ml/min)	97.05 (87.84–104.92)	97.07 (85.96–106.15)	97.28 (86.43–105.26)	95.68 (82.05–105.04)	.264
Uric acid (umol/L)	341.40 (286.95–402.65)	354.80 (294.50–419.85)	351.30 (294.40–427.00)	359.30 (296.00–420.90)	.058
FBG (mmol/L)	6.25 (5.31–8.25)	6.47 (5.47–8.57)	6.47 (5.51–8.81)	6.80 (5.54–9.36)	.003
HbA1c (%)	6.00 (5.60–7.20)	6.00 (5.60–7.40)	6.10 (5.70–7.30)	6.10 (5.60–7.40)	.258
TC (mmol/L)	3.87 (3.19–4.61)	4.12 (3.49–4.93)	4.13 (3.54–4.92)	4.35 (3.63–5.11)	<.001
TG (mmol/L)	1.39 (0.98–1.93)	1.63 (1.20–2.27)	1.53 (1.13–2.16)	1.52 (1.14–2.20)	<.001
LDL‐C (mmol/L)	2.24 (1.74–2.89)	2.46 (1.95–3.12)	2.55 (2.05–3.19)	2.77 (2.17–3.34)	<.001
HDL‐C (mmol/L)	1.02 (0.88–1.18)	0.97 (0.83–1.12)	0.93 (0.82–1.09)	0.94 (0.82–1.08)	<.001
CK‐MB (ng/L)	1.90 (1.20–9.95)	2.70 (1.40–30.05)	3.60 (1.50–78.50)	10.40 (2.10–99.30)	<.001
hs‐TnI (ng/L)	0.12 (0.10–1.35)	0.43 (0.05–3.28)	1.05 (0.12–8.44)	4.21 (0.52–17.12)	<.001
Lesion charateristic					
LM disease	35 (7.7)	28 (6.2)	31 (6.9)	34 (7.5)	.815
One‐vessel disease, *n* (%)	114 (25.2)	108 (24.1)	106 (23.5)	104 (23.1)	.893
Two‐vessel disease, *n* (%)	149 (32.9)	168 (37.4)	155 (34.4)	147 (32.6)	.405
Three‐vessel disease, *n* (%)	185 (40.8)	171 (38.1)	185 (41.0)	196 (43.5)	.441
Target vessel territory					
LAD, *n* (%)	237 (52.3)	213 (47.4)	226 (50.1)	235 (52.1)	.428
LCX, *n* (%)	101 (22.3)	120 (26.7)	115 (25.5)	130 (28.8)	.152
RCA, *n* (%)	173 (38.2)	185 (41.2)	171 (37.9)	146 (32.4)	.050
Clinical diagnosis					
STEMI, *n* (%)	223 (49.2)	235 (52.3)	232 (51.4)	254 (56.3)	.190
NSTEMI, *n* (%)	230 (50.8)	214 (47.7)	219 (48.6)	197 (43.7)	.190
Medications in hospital					
Aspirin, *n* (%)	452 (99.8)	447 (99.6)	449 (99.6)	451 (100)	.529
Clopidogrel/ticagrelor, *n* (%)	452 (99.8)	447 (99.6)	449 (99.6)	450 (99.8)	.878
Statin, *n* (%)	452 (99.8)	449 (100)	451 (100)	450 (99.8)	.574
ACEI/ARB, *n* (%)	192 (42.4)	193 (43.0)	205 (45.5)	222 (49.2)	.153
β‐Blockers, *n* (%)	358 (79.0)	337 (75.1)	348 (77.2)	368 (81.6)	.106
CCB, *n* (%)	68 (15.0)	68 (15.1)	63 (14.0)	47 (10.4)	.133
Nitrate, *n* (%)	418 (92.3)	407 (90.6)	416 (92.2)	410 (90.9)	.731
IIbIIIA, *n* (%)	52 (11.5)	59 (13.1)	60 (13.3)	83 (18.4)	.018

*Note*: Values are presented as the mean ± SD, median (IQR), or number (%).

Abbreviations: ACEI, angiotensin‐converting enzyme inhibitor; ARB, angiotensin receptor blocker; BMI, body mass index; BP, blood pressure; CABG, coronary artery bypass grafting; CCB, calcium channel blockers; CK‐MB, creatine kinase isoenzyme‐MB; eGFR, estimated glomerular filtration rate; FBG, fasting blood glucose; Grace, Global Registry of Acute Coronary Events; HbA1c, glycosylated hemoglobin A1c; HDL‐C, high‐density lipoprotein cholesterol; hs‐CRP, high‐sensitivity C‐reactive protein; hs‐TnI, high sensitive troponin I; IIbIIIA, IIBbIIIA receptor antagonist; IQR, interquartile range; LAD, left anterior descending artery; LCX, left circumflex artery; LDL‐C, low‐density lipoprotein cholesterol; LM, left main; LVEF, left ventricular ejection fraction; MI, myocardial infarction; NSTEMI, non ST‐segment elevation myocardial infarction; PCI, percutaneous coronary intervention; RCA, right coronary artery; SCr, serum creatinine; STEMI, ST‐segment elevation myocardial infarction; TC, total cholesterol; TG, triglycerides; WBC, white blood cell.

### 3.2 hs‐CRP and the in‐hospital outcome

The relationship between serum hs‐CRP and in‐hospital outcomes were shown in Table S1. In‐hospital mortality comes about 6 (0.3%) patients. Malignant arrhythmia comes from 29 (1.6%) patients. Mechanical ventilation comes about 76 (4.2%) patients. Congestive HF comes about 284 (15.7%) patients. Cardiogenic shock come about 37 (2.1%) patients. Thrombosis comes about 20 (1.1%) patients. Stroke come about 195 (10.8%) patients. BARC bleeding ≥ 2 comes about 26 (1.4%) patients. A total of 259 (66%) hospitalization outcomes were recorded in the third and fourth quartiles, with 179 patients in the fourth quartiles, in which mortality was significantly higher than the other quartiles (*p* = .011). Interestingly, malignant arrhythmia, mechanical complication, congestive HF, cardiogenic shock, BARC bleeding ≥ 2, and in‐hospital combined outcomes show a significant trend increase (*p* < .01 for trend).

### 3.3 hs‐CRP as an independent predictor of in‐hospital outcome occurrence

The univariate logistic regression analysis showed that age, systolic BP, diastolic BP, heart rate, hypertension, diabetes, previous stroke, LVEF, Grace score, hs‐CRP, WBC count, hemoglobin, SCr, estimated glomerular filtration rate, uric acid, FBG, glycosylated hemoglobin A1c, triglycerides, creatine kinase isoenzyme‐MB, hs‐TnI, left main disease, Three‐vessel disease, left anterior descending artery, left circumflex artery, STEMI, angiotensin‐converting enzyme inhibitor/angiotensin receptor blocker, β‐blockers, IIbIIIa receptor antagonists were risk factor prognostic indicator for the in‐hospital outcome (Table S2). To further clarify whether Grace score, hs‐CRP was associated with in‐hospital outcome events, we put significant variables (*p* < .05) in univariate analysis into the multivariate logistic regression analysis, the results show that Grace score, hs‐CRP were still a significant independent risk factor for outcome events, as shown in Table 2.

**Table 2:** Multivariate logistic regression analysis for predictors of in‐hospital outcome

Variable	OR	95% CI	*p*‐Value
Hypertension	1.095	0.800–1.497	.571
Diabetes	1.342	0.912–1.974	.136
Previous stroke	2.722	1.808–4.098	<.001
LVEF (%)	0.943	0.926–0.960	<.001
Grace score	1.042	1.035–1.049	<.001
hs‐CRP (mg/L)	1.100	1.076–1.124	<.001
WBC count (×10^9^/L)	1.067	1.011–1.125	.017
Hemoglobin (g/L)	1.001	0.991–1.010	.91
Uric acid (umol/L)	1.001	1.000–1.003	.188
FBG (mmol/L)	0.989	0.933–1.048	.713
HbA1c (%)	1.063	0.934–1.209	.358
TG (mmol/L)	0.997	0.891–1.116	.962
LM disease	1.283	0.758–2.172	.353
Three‐vessel disease	1.013	0.751–1.366	.933
LAD	1.391	1.025–1.889	.034
LCX	1.077	0.753–1.540	.685
STEMI	2.208	1.603–3.041	<.001
ACEI/ARB	1.545	1.143–2.088	.005
β‐Blockers	1.132	0.777–1.649	.519
IIbIIIA	1.430	0.966–2.116	.074

Abbreviations: ACEI, angiotensin‐converting enzyme inhibitor; ARB, angiotensin receptor blocker; CI, confidence interval; FBG, fasting blood glucose; Grace, Global Registry of Acute Coronary Events; HbA1c, glycosylated hemoglobin A1c; hs‐CRP high‐sensitivity C‐reactive protein; IIbIIIA, IIBbIIIA receptor antagonist; LAD, left anterior descending artery; LCX, left circumflex artery; LM, left main; LVEF, left ventricular ejection fraction; OR, odds ratio; STEMI, ST‐segment elevation myocardial infarction; TC, total cholesterol; TG, triglycerides; WBC, white blood cell.

### 3.4 Association and combination of GRACE risk score with admission serum hs‐CRP

**Figure 1:**
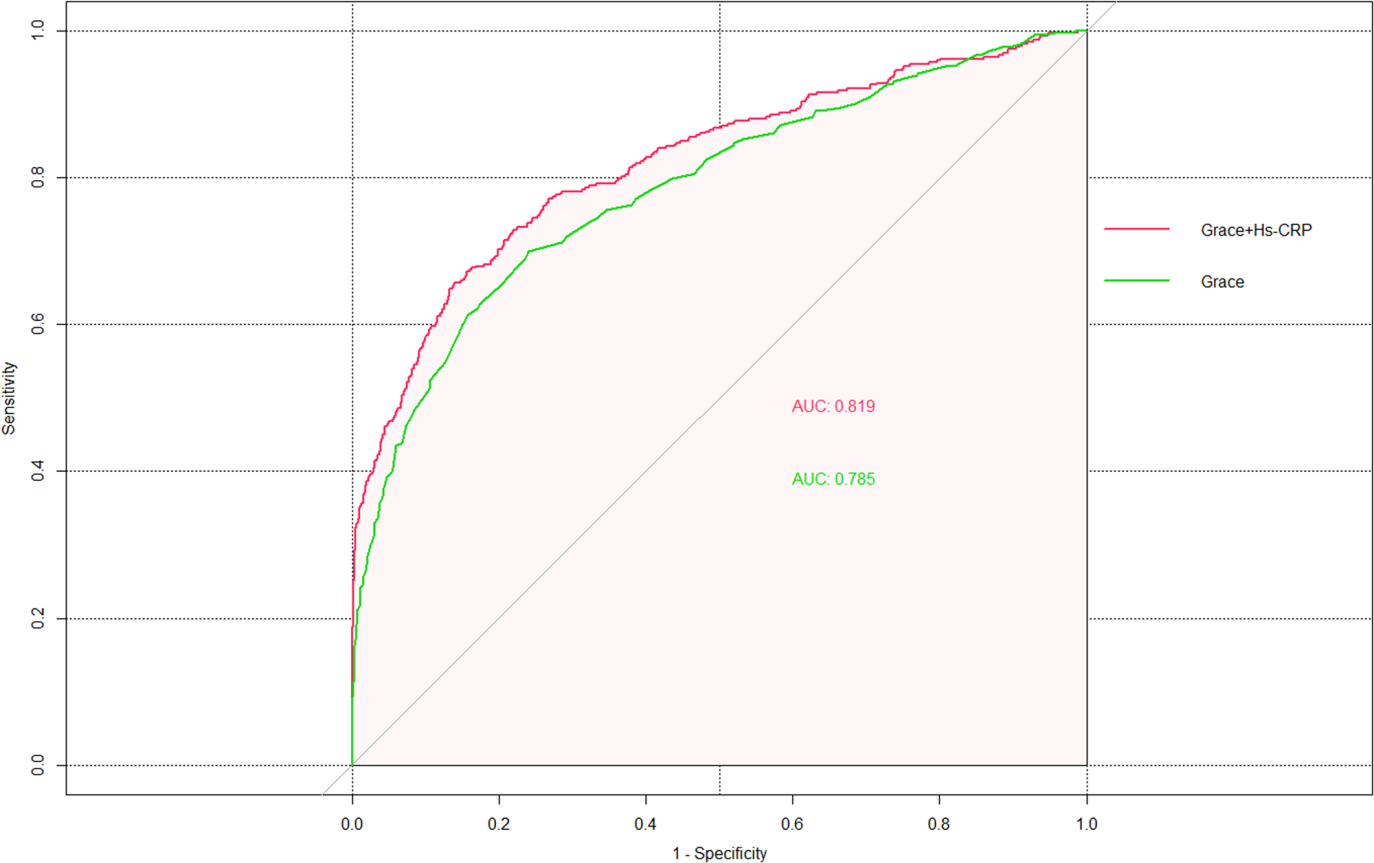
The receiver operating characteristic (ROC) curves comparing the Grace risk score and its combination with admission serum hs‐CRP. (The area under the ROC curve [AUC] for  Grace + hs‐CRP score was 0.819; Grace risk score alone was 0.785; [the difference in the AUC was 0.034, *Z* = 4.145, *p* < .001]). Grace, Global Registry of Acute Coronary Events; hs‐CRP, high‐sensitivity C‐reactive protein

**Figure 2:**
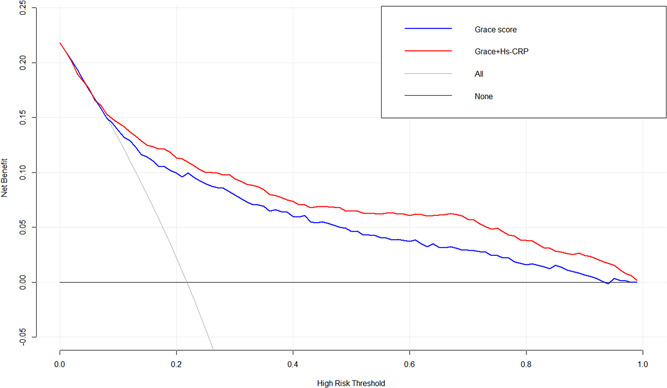
The decision curve analysis (DCA) comparing the Grace risk score and its combination with admission serum hs‐CRP. (The clinical benefit of the Grace + hs‐CRP model was significantly higher than the traditional Grace model). Grace, Global Registry of Acute Coronary Events; hs‐CRP, high‐sensitivity C‐reactive protein

The relation between hs‐CRP and Grace risk score was estimated by Spearman correlation analysis, the result showed that the hs‐CRP and Grace risk score had a significantly positive correlation (*r* = .191, *p* < .001). Multivariate logistic regression analysis confirmed that both hs‐CPR and Grace risk score were independent risk factors for predicting in‐hospital outcome events, so we used several methods to further evaluate the ability of hs‐CRP combined with Grace risk score to predict hospitalization outcome events. First, we evaluated the differentiation of the new model. The ROC curve showed that the ACU area of hs‐CRP combined with the Grace risk score model significantly increased from 0.785 of the traditional GRACE model alone to 0.819 (the difference in the AUC was 0.034, *Z* = 4.145, *p* < .001; Figure 1). We calculated the Youden index corresponding to the optimal threshold for hs‐CRP to predict in‐hospital outcomes as the cut point to calculate NRI. This cut‐point value is 0.287, thus we used 28.7% as the arbitrary thresholds to define low and high risk. The NRI for the new risk model was 0.05956 (*p* = .007), with events contributing 0.04326 (*p* = .026) and nonevents 0.01630 (*p* = .108), indicating that the new model could improve the ability to correctly redistinguish the occurrence of hospitalization outcome events. The IDI for the new model was 0.0757 (95% CI: 0.0573–0.0942, *p* < .001) again shows significant improvement in the diagnostic performance of the new model, as shown in Table 3. Among 393 patients of the group with the occurrence of outcome events in this study, 27 patients in the high‐risk group predicted by the old model were wrongly assigned to the low‐risk group by the new model. The old model predicted that 108 in the low‐risk group would be correctly reassigned to the high‐risk group by the new model. However, in the 1411 patients with nonevents group, 68 patients in the low‐risk group predicted by the old model were wrongly assigned to the high‐risk group by the new model, and 91 patients in the high‐risk group predicted by the old model were correctly assigned to the low‐risk group by the new model, as shown in Table S3. Second, we evaluated the calibration degree of the model (Figure S1). It can be seen that the calibration plot of the GRACE model alone and the new model are very close to the theoretical curves, indicating that the prediction and actual events show good overall consistency. Finally, we conducted a clinical effectiveness evaluation, and the results showed that the clinical benefit of the new model was significantly higher than the traditional Grace model (Figure 2).

**Table 3:** Statistics for model improvement with the addition of hs‐CRP content

		*p*‐Value
Events, *n* (%)	393 (21.8)	
Nonevents, *n* (%)	1411 (78.2)	
Categorical NRI (%)		
cNRI event	4.326	.026
cNRI nonevent	1.630	.108
cNRI	5.956	.007
IDI statistics		
IDI	0.0757 (95% CI: 0.0573–0.0942)	<.001
AUC		
Grace risk score	0.785 (95% CI: 0.758‐0.813)	
Grace + hs‐CRP	0.819 (95% CI: 0.793‐0.845)	
Difference	0.034	<.001

Abbreviations: 95% CI, 95% confidence interval; cNRI, categorical net reclassification improvement; Grace, Global Registry of Acute Coronary Events; hs‐CRP, high‐sensitivity C‐reactive protein; IDI, integrated discrimination improvement; NRI, net reclassification improvement.

## 4 DISCUSSION

In this study, our main findings include: (1) a high level of admission hs‐CRP is a significant independent risk factor for in‐hospital outcome events in patients with AMI. (2) There was a significant positive correlation between hs‐CRP and Grace risk score, and the inclusion of hs‐CRP in the Grace risk score could improve the ability to correctly distinguish the occurrence of in‐hospital outcomes.

Inflammation plays a key role in atherosclerosis, plaque rupture, and thrombosis,^10^ which has been considered as one of the important risk factors of cardiovascular events.^4^ hs‐CRP is one of the biomarkers of inflammation, which has been proved to be associated with the risk of atherosclerotic cardiovascular disease.^5^ Meanwhile, statins have also been shown to have anti‐inflammatory effects in addition to lowering cholesterol.^11^ The results of observation and randomized controlled trials showed that patients with cardiovascular disease benefit more significantly from the lower systemic inflammatory response. All these suggest that inflammation is involved in the process of AMI, but it is not clear whether CRP is related to complications after myocardial infarction.

Many previous studies have shown that CRP is associated with the long‐term prognosis of AMI. Xia et al.^12^ estimated the association between CRP levels and long‐term all‐cause, cardiovascular, and cardiac mortality in AMI patients showed that CRP value is an independent predictor of long‐term cardiac mortality after AMI. The prospective study from Suleiman et al.^13^ enrolled 1044 AMI patients to investigate the relationship between CRP and long‐term risk of death, HF in survivors of AMI, the results suggested that C‐reactive protein is a marker of long‐term development of HF and mortality in patients with AMI. Unlike previous studies, the present study focused on the relationship between hs‐CRP and short‐term outcome events during hospitalization after AMI. These adverse hospital outcomes are closely associated with common complications in patients with AMI (i.e., in‐hospital death, malignant arrhythmia, mechanical complication, congestive HF, cardiogenic shock, bleeding, and stroke). In our study, the highest quartile of hs‐CRP was related to in‐hospital events such as death, HF, cardiogenic shock, bleeding, and mechanical complications. At the same time, after a multivariate logistic regression analysis to exclude potential confounders, the results showed that hs‐CRP was still a risk factor for hospitalization combined events. Our results were supported those of previous research. For example, we found the highest admission level of hs‐CRP was significantly associated with congestive HF, the result is consistent with the previous studies, such as the study from Stumpf et al.^14^ showed that peak CRP is a strong predictor of HF and cardiovascular mortality in STEIMI patients. Moreover, we performed that high‐level hs‐CRP also correlated with early mechanical complications, our finding in line with the previous research by Anzai et al.^15^ This study included 220 patients with a first Q‐wave AMI to investigate the relationship between CRP and cardiac rupture in patients with AMI, the result showed that elevation of CRP levels after AMI may predict cardiac rupture, left ventricular aneurysmal formation, and infarct expansion. Interestingly, we found that these in‐hospital adverse events increased significantly with the increase of hs‐CRP concentration (*p* for trend < .001). On the other hand, our results inconsistent with those of other studies. Such as we found hs‐CRP was not related to thrombosis, but the study from Anzai et al.^16^ showed that levels of hs‐CRP are predictors to indicate thrombus formation. I think it may be due to our short observation period and fewer cases. The mechanism of hs‐CRP in AMI patients is not only related to inflammation, but also related to different organ damage caused by oxidative stress, endothelial dysfunction, and mitochondrial abnormalities.^17^, ^18^, ^19^

The Grace risk score has been widely recommended globally for risk stratification in patients with AMI to assess short‐term (in‐hospital or 30 days) and medium‐ to long‐term (≥1 year) outcomes.^3^ The Grace score system includes two biological indicators, including SCr and myocardial enzymes, which indicates that these biological indicators can reflect the pathophysiological process of myocardial infarction. Previous studies have added other new biological indicators to the Grace score system for predicting adverse clinical outcomes in patients with ACS, these novel biological indicators are NT‐proBNP,^20^ platelet reactivity,^21^ serum calcium levels,^22^ serum acid uric levels,^23^ RDW/PDW,^24^ and other factors. The results showed that these biomarkers in the Grace score may improve the identification of clinical outcomes in ACS patients, However, it is not clear whether hs‐CRP as a nontraditional risk of AMI also enhances the ability of Grace risk stratification. In our study, the ACU area of hs‐CRP combined with the Grace risk score model significantly increased from 0.785 of the traditional Grace model alone to 0.819, the difference was statistically significant. Moreover, we found hs‐CRP combined Grace score model could improve the ability to correctly redistinguish the occurrence of hospitalization outcome events (the INR was 5.96%; *p* = .007). On the other hand, we found the new model of the prediction and actual events show good overall consistency. Finally, we found that the clinical benefit of the new model was significantly higher than the traditional Grace model. Therefore, our finding could support the assumption that the hs‐CRP combined Grace risk score model enhances the ability of the Grace score system to predict the risk of in‐hospital outcomes in patients with AMI.

## 5 LIMITATION

This study had several limitations. First, this study was a single‐center, retrospective study, which may have a selection bias or potential confounding factors. Second, we did not exclude patients with systemic inflammatory response, including acute infection, which may affect the concentration of hs‐CRP. Third, the biochemical parameters were only measured at admission, and the dynamic changes of hs‐CRP were not monitored during the in‐hospital, which may result in measurement bias.

## 6 CONCLUSION

Admission hs‐CRP level was a significant independent risk factor for in‐hospital outcome events in patients with AMI. The inclusion of hs‐CRP in the Grace risk score could improve the ability to correctly distinguish the occurrence of in‐hospital outcomes. Further prospective, large, multicenter studies are needed to confirm our findings.

## CONFLICT OF INTERESTS

The authors declare that there are no conflict of interests.

## Supporting information


**Figure S1:** The calibration plot of the Grace risk score and its combination with admission serum hs‐CRP. (The two calibration plot are very close to the theoretical curves, indicating that the prediction and actual events show good overall consistency).

Supporting information.

## Data Availability

The data that support the findings of this study are available on request from the corresponding author.
