# Supplementary figures and images for: Admission high‐sensitivity C‐reactive protein levels improve the Grace risk score prediction on in‐hospital outcomes in acute myocardial infarction patients
Source: Clin Cardiol. 2022 Jan 23;45(3):282–90. doi: 10.1002/clc.23749 (PMC8922537; doi:10.1002/clc.23749)

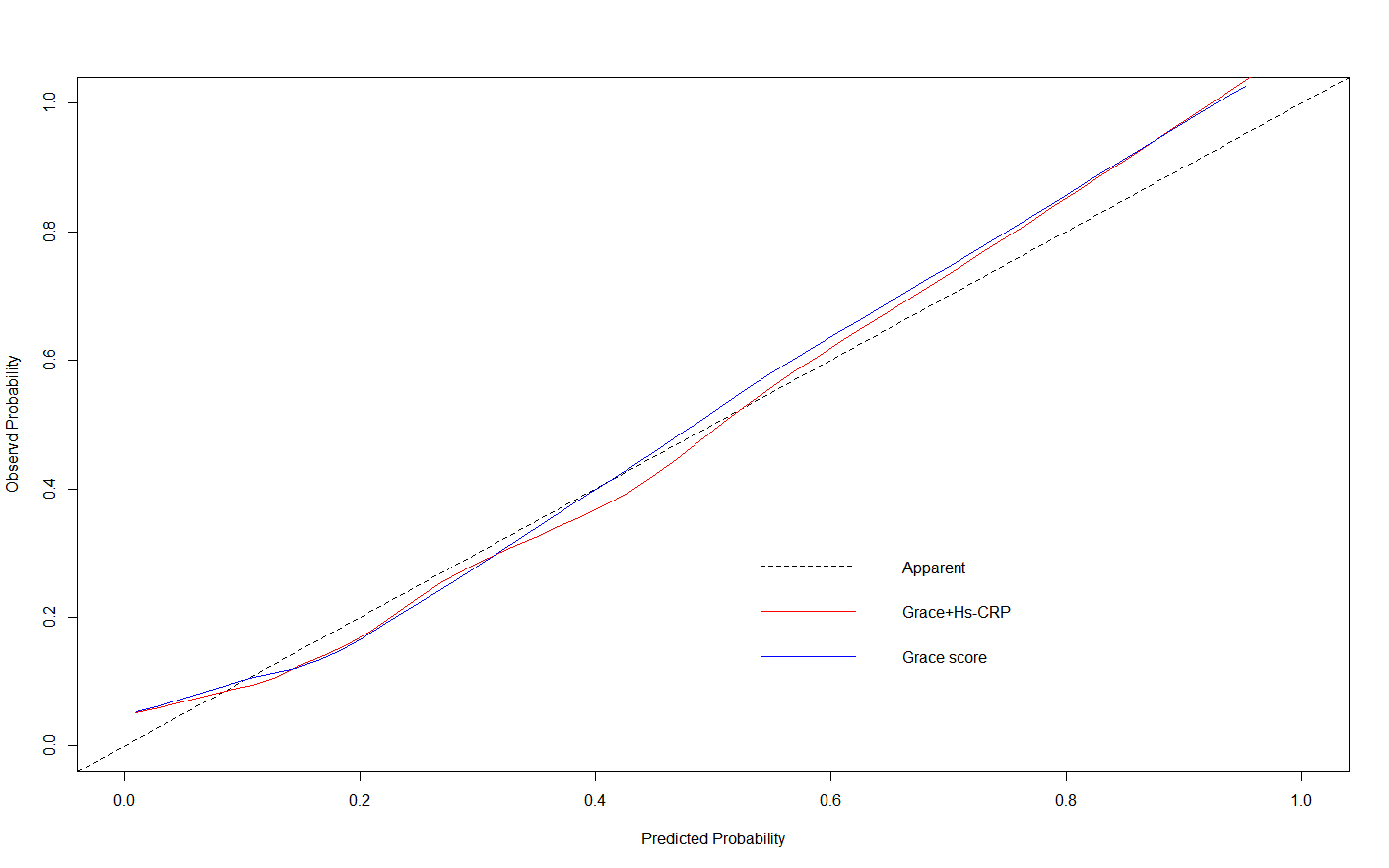

Supplement: Supplementary file 1 — Figure S1: The calibration plot of the Grace risk score and its combination with admission serum hs‐CRP. (The two calibration plot are very close to the theoretical curves, indicating that the prediction and actual events show good overall consistency). [file CLC-45-282-s002.tiff]
