# Supplementary material for: Admission high‐sensitivity C‐reactive protein levels improve the Grace risk score prediction on in‐hospital outcomes in acute myocardial infarction patients
Source: Clin Cardiol. 2022 Jan 23;45(3):282–90. doi: 10.1002/clc.23749 (PMC8922537; doi:10.1002/clc.23749)
Supplement: Supplementary file 2 — Supporting information. [file CLC-45-282-s001.docx]

**Table S1 In-hospital outcomes according by quartiles of hs-CRP**

|  | Hs-CRP quartiles | | | | |
| --- | --- | --- | --- | --- | --- |
|  | Q1  (<1.23 mg/L) | Q2  (1.23-3.02 mg/L) | Q3  (3.02-7.80 mg/L) | Q4  (≥7.80 mg/L) | P value |
| Death, n (%) | 1 (0.2) | 0 (0) | 0 (0) | 6 (1.3) | 0.011 |
| Malignant arrhythmia, n (%) | 5 (1.1) | 5 (1.1) | 6 (1.3) | 13 (2.9) | 0.036 |
| Mechanical complication, n (%) | 8 (1.8) | 10 (2.2) | 18 (4.0) | 40 (8.9) | <0.001 |
| Congestive heart failure, n (%) | 43 (9.5) | 45 (10.0) | 49 (13.1) | 137 (30.4) | <0.001 |
| Cardiogenic shock, n (%) | 5 (1.1) | 4 (0.9) | 7 (1.6) | 21 (4.7) | <0.001 |
| Thombosis, n (%) | 7 (1.5) | 1 (0.1) | 4 (0.2) | 8 (0.4) | 0.544 |
| BARC bleeding≥2, n (%) | 4 (0.9) | 4 (0.9) | 5 (1.1) | 13 (2.9) | 0.013 |
| Stroke, n (%) | 8 (1.8) | 11 (2.4) | 6 (1.3) | 10 (2.2) | 0.935 |
| In-hospital combined outcomes, n (%) | 66 (14.6) | 68 (15.1) | 80 (17.7) | 179 (39.7) | <0.001 |

**Table S2 Univariate and Multivariate Logistic Regression Analysis for Predictors of In-hospital outcome**

| Variable | OR | 95% CI | P Value |
| --- | --- | --- | --- |
| Age,years | 1.036 | 1.025-1.047 | <0.001 |
| Male Sex | 0.813 | 0.619-1.067 | 0.135 |
| BMI,kg/m² | 0.975 | 0.944-1.007 | 0.130 |
| Systolic BP, mmHg | 0.986 | 0.980-0.993 | <0.001 |
| Diastolic BP, mmHg | 0.982 | 0.972-0.992 | <0.001 |
| Heart rate, bpm | 1.022 | 1.013-1.031 | <0.001 |
| Hypertension | 1.272 | 1.00-1.604 | 0.043 |
| Diabetes | 1.627 | 1.287-2.049 | <0.001 |
| Dyslipidemia | 0.824 | 0.656-1.034 | 0.095 |
| Previous or current Smoking | 0.818 | 0.651-1.026 | 0.083 |
| Previous MI | 0.844 | 0.613-1.163 | 0.300 |
| Previous PCI | 0.890 | 0.579-1.368 | 0.594 |
| Previous CABG | 0.796 | 0.268-2.365 | 0.681 |
| Previous Stroke | 2.471 | 1.805-3.383 | <0.001 |
| LVEF (%) | 0.907 | 0.894-0.920 | <0.001 |
| GRACE score | 1.045 | 1.040-1.051 | <0.001 |
| Hs-CRP mg/L | 1.134 | 1.114-1.153 | <0.001 |
| Laboratory values at hospital admission |  |  |  |
| WBC count, ×10^9^/L | 1.134 | 1.091-1.179 | <0.001 |
| Hemoglobin, g/L | 0.977 | 0.970-0.984 | <0.001 |
| Platelet count, ×10^9^/L | 0.999 | 0.998-1.001 | 0.557 |
| SCr, mmol/L | 1.007 | 1.004-1.011 | <0.001 |
| eGFR, mL/min | 0.978 | 0.973-0.984 | <0.001 |

**Table S2 (continued)**

| Variable | OR | 95% CI | P Value |
| --- | --- | --- | --- |
| Uric acid, umol/L | 1.001 | 1.000-1.002 | 0.035 |
| FBG, mmol/L | 1.100 | 1.067-1.135 | <0.001 |
| HbA1c, % | 1.144 | 1.070-1.223 | <0.001 |
| TC (mmol/L) | 0.926 | 0.836-1.025 | 0.138 |
| TG (mmol/L) | 0.875 | 0.787-0.972 | 0.012 |
| LDL-C (mmol/L) | 0.967 | 0.857-1.091 | 0.585 |
| HDL-C (mmol/L) | 0.827 | 0.507-1.349 | 0.447 |
| CK-MB (ng/L) | 1.003 | 1.002-1.004 | <0.001 |
| hs-TnI (ng/L) | 1.016 | 1.011-1.021 | <0.001 |
| Lesion charateristic |  |  |  |
| LM disease | 2.069 | 1.413-3.030 | <0.001 |
| One-vessel disease | 0.769 | 0.585-1.011 | 0.060 |
| Two-vessel disease | 0.879 | 0.692-1.115 | 0.288 |
| Three-vessel disease | 1.348 | 1.76-1.688 | 0.009 |
| Target vessel territory |  |  |  |
| LAD | 1.617 | 1.289-2.209 | <0.001 |
| LCX | 0.732 | 0.560-0.958 | 0.023 |
| RCA | 0.797 | 0.630-1.009 | 0.059 |
| Clinical diagnosis |  |  |  |
| NSTEMI | Ref |  |  |
| STEMI | 2.232 | 1.764-2.824 | <0.001 |
| Medications in hospital |  |  |  |
| Aspirin | 2.401 | 0.400-14.418 | 0.338 |

**Table S2 (continued)**

| Variable | OR | 95% CI | P Value |
| --- | --- | --- | --- |
| Clopidogrel/Ticagrelor | 0.556 | 0.101-3.046 | 0.499 |
| Statin | 3.597 | 0.224-57.636 | 0.366 |
| ACEI/ARB | 1.713 | 1.368-2.146 | <0.001 |
| β-blockers | 1.528 | 1.140-2.049 | 0.005 |
| CCB | 0.708 | 0.498-1.007 | 0.055 |
| Nitrate | 1.014 | 0.678-1.517 | 0.946 |
| IIbIIIA | 1.48 | 1.096-1.998 | 0.011 |

Abbreviations: BMI, body mass index; MI, myocardial infarction; PCI, percutaneous coronary intervention; CABG, coronary artery bypass grafting; LVEF, left ventricular ejection fraction; hs-CRP High-sensitivity C-reactive protein; WBC, white blood cell; SCr, serum creatinine; eGFR, estimated glomerular filtration rate; FBG, fasting blood glucose; HbA1c, glycosylated hemoglobin A1c; TC, total cholesterol; TG, triglycerides; LDL-C, low-density lipoprotein cholesterol; HDL-C, high-density lipoprotein cholesterol; CK-MB, Creatine Kinase Isoenzyme-MB; hs-TnI，high sensitive troponin I；LM, left main; LAD, left anterior descending artery; LCX, left circumflex artery; RCA, right coronary artery；STEMI, ST-segment elevation myocardial infarction; NSTEMI, non ST-segment elevation myocardial infarction; ACEI, angiotensin converting enzyme inhibitor; ARB, angiotensin receptor blocker; CCB, Calcium Channel Blockers; IIbIIIA, IIBbIIIA receptor antagonist; OR, odds ratio; 95 % CI, 95 % confidence interval.

**Table S3. Reclassification among people who experience in-hospital outcomes and those who do not experience**

| Events group | hs-CRP+Grace model (new) |  | Total |
| --- | --- | --- | --- |
| Grace model (old) | ＜0.287 | ≥0.287 |  |
| ＜0.287 | 108 | 44 | 152 |
| ≥0.287 | 27 | 214 | 241 |
| Total | 135 | 258 | 393 |
| Non Events group | | | |
| ＜0.287 | 1120 | 68 | 1188 |
| ≥0.287 | 91 | 132 | 223 |
| Total | 1211 | 200 | 1411 |
